# Comparative risk assessment of non-communicable diseases by evacuation scenario– a retrospective study in the 7 years following the Fukushima Daiichi nuclear power plant accident

**DOI:** 10.1080/16549716.2021.1918886

**Published:** 2021-06-01

**Authors:** Shuhei Nomura, Michio Murakami, Akihiko Ozaki, Toyoaki Sawano, Claire Leppold, Yoshitaka Nishikawa, Hiroaki Saito, Tomoyoshi Oikawa, Masaharu Tsubokura

**Affiliations:** a Research Center for Community Health, Minamisoma Municipal General Hospital, Fukushima, Japan; b Department of Global Health Policy, Graduate School of Medicine, The University of Tokyo, Tokyo, Japan; c Department of Health Policy and Management, School of Medicine, Keio University, Tokyo, Japan; d Department of Health Risk Communication, Fukushima Medical University School of Medicine, Fukushima, Japan; e Department of Breast Surgery, Jyoban Hospital of Tokiwa Foundation, Fukushima, Japan; f Department of Surgery, Jyoban Hospital of Tokiwa Foundation, Fukushima, Japan; g Department of Radiation Health Management, Fukushima Medical University School of Medicine, Fukushima, Japan; h Child and Community Wellbeing Unit, Centre for Health Equity, Melbourne School of Population and Global Health, University of Melbourne, Melbourne, Australia; i Department of Internal Medicine, Soma Central Hospital, Fukushima, Japan; j Department of Health Informatics, School of Public Health, Kyoto University, Kyoto, Japan; k Department of Gastroenterology, Sendai Kousei Hospital, Miyagi, Japan; § Department of Neurosurgery, Minamisoma Municipal General Hospital, Fukushima, Japan

**Keywords:** Japan, Fukushima, evacuation, non-communicable diseases

## Abstract

**Background**: As a result of the Fukushima Daiichi nuclear power plant accident, many residents evacuated and were exposed to changes in their living environment and socioeconomic status, and to persistent stressors. Past studies have suggested the potential for these circumstances to contribute to long-term changes to population health.

**Objective**: The objective of this study was to gain a better understanding of long-term health effects of evacuation, by evaluating the risk of non-communicable diseases among evacuees from Minamisoma City (one of the closest municipalities to the power plant) until 2017.

**Methods**: The study evaluated data from annual health check-ups for residents aged 40–74 years covered by National Health Insurance (who are largely self-employed) from 2010 to 2017 administered by Minamisoma City. Diabetes, hyperlipidemia, and hypertension were defined from the results of blood sampling. Annual changes in age-adjusted prevalence were estimated by evacuation scenario. We also performed an inverse-probability weighting (IPW) analysis to adjust for baseline covariates in 2010 and estimated the differences in the risk of diabetes, hyperlipidemia, and hypertension by evacuation scenario as of the 2017 health check-up in reference to the no-evacuation group.

**Results**: A total of 1,837 individuals were considered in this study. Regardless of evacuation scenario, there was statistical evidence suggesting an upward and a downward trend in diabetes and hypertension from 2010 to 2017, respectively, while hyperlipidemia showed no remarkable change. IPW analyses demonstrated that disease risks in 2017 did not differ significantly among people with different evacuation scenarios.

**Conclusions**: Region-specific factors played an important role in the health effects of the evacuation. Our findings have important implications for the need of an assessment of the health effects of evacuations in more localized manner. Further research in this area will strengthen the communities’ preparedness for future disasters that require mass evacuation.

## Main texts

### Background

On 11 March 2011, the Great East Japan Earthquake caused a tsunami and subsequent nuclear accident at Fukushima Daiichi nuclear power plant, releasing radioactive materials into the environment, particularly over a wide area in east Japan. At the time of the nuclear accident, cumulative radiation doses via external and internal exposure to radionuclides were of great public health concern. However, continuous assessment of radiation exposure levels from the Fukushima nuclear accident by international bodies and academic researchers suggest that the doses received in the general public in the first year of the accident, and the estimates for their lifetimes, are generally low [1–6] and associated with a very low likelihood of physical health effects [7,8].

However, the health effects of the Fukushima nuclear accident are not limited to those directly caused by radiation exposure [9,10]. Major disasters including natural events are often followed by human evacuation, which can have an impact on individual vulnerability to persistent psychological stress and changes in socioeconomic status (e.g. unemployment, reduced incomes, and living in shelters, temporary housing, and rental housing) [11]. As a result, evacuation can affect population health. By 29 August 2011, more than 140 thousand people had been forced to evacuate after the Fukushima nuclear accident [12]. Some moved elsewhere within Fukushima Prefecture, and some moved out of this prefecture. Evacuation sites included shelters, temporary housing, neighbours/relatives house, or new houses. Previous studies have identified possible health risks associated with evacuation following the accident [13–17]. Nomura et al. (2016) suggested the possibility that evacuation was related to the increased risk of non-communicable diseases (including diabetes, hyperlipidemia, and hypertension) in the first two years after the accident in Minamisoma City, one of the municipalities in the closest to the nuclear power plant in Fukushima Prefecture [14]. In another municipality where all residents were evacuated, the results of a 3-year follow-up of the evacuees showed an increase in the prevalence of diabetes, hyperlipidemia, and hypertension compared to the pre-accident period [17]. Satoh et al. (2015) also followed Fukushima residents and suggested a relationship between evacuation and increased risk of diabetes [15].

There has been repeated emphasis that it is important to continue follow-up studies in order to understand the health status of evacuees, to prevent, detect, and treat diseases at an early stage, and to maintain and improve the health of residents in the future [18–21]. The Sendai Framework for Disaster Risk Reduction 2015–2030 (the 2015 United Nations landmark agreements for disaster risk reduction) also points out the importance of post-disaster follow-up in its Priority 4 (‘Enhancing disaster preparedness for effective response and to “Build Back Better” in recovery, rehabilitation and reconstruction’). Long-term follow-up can provide valuable information for public health systems and ultimately enhance disaster preparedness [22]. Meanwhile, nine years after the nuclear accident, few studies have tracked evacuees over the long-term to assess their health to date. Evacuation scenarios, that is, the timing of returning from short- or long-term evacuation, can differ person to person. In this study, we followed evacuees and non-evacuees from Minamisoma City from 2010 to 2017, and evaluated differences in the risk of non-communicable diseases by different evacuation scenarios. The purpose of this study was to gain a greater understanding of any long-term health effects of evacuation in areas affected by the Fukushima nuclear accident, to provide insights that will contribute to future disaster preparedness that require mass evacuation.

## Methods

### Setting

The study area is Minamisoma City, Fukushima Prefecture, Japan. On 12 March 2011, the 20 km radius around the Fukushima Daiichi nuclear power plant was designated as a mandatory evacuation area. Part of Minamisoma City was included in this mandatory evacuation area, where more than 14,000 people, equivalent to 20% of the total population of Minamisoma City lived as that time [23]. Minamisoma City had a pre-accident population of 71,561 as of 11 March 2011 [24]. On 22 April 2011, the mandatory evacuation area was slightly expanded to the northwest direction based on the measurement of the dispersion of high-concentration radioactive fallout. As time has passed since the nuclear accident, evacuation orders have been lifted one by one at the sub-regional level, corresponding to a decrease in air dose radiation levels [25]. In Minamisoma City, most of the evacuation area have been lifted except for some local areas where there are a few households [23]. It is important to note that many people outside the evacuation area also voluntarily evacuated after the accident for other reasons (e.g. concerns about radiation exposure as well as inconvenience of their lives due to the designation of their neighborhoods as an evacuation are). For example, as of 29 February 2020, the population of Minamisoma City was 59,717 according to the resident register, but 4,955 people lived outside the city [26]. In other words, as is often the case after a disaster, the address on the certificate of residence and actual place of residence may differ (see below). The geographical location of Minamisoma City and the temporal changes in the setting of the evacuation area can be found elsewhere [14,27].

### Data collection and description

#### Participants

This study focuses on the long-term health assessment in the areas affected by the accident. For this purpose, this study includes those who participated in the health check-ups in 2010 (first year of study) and 2017 (last year of study). The study participants were adults aged 40–74 years, who were eligible for National Health Insurance (NHI) health check-ups (described below).

#### Data – health check-ups

There are three main types of health insurance in Japan: Employee Health Insurance (EHI), NHI, and Late Elders’ Health Insurance (LEHI) [28]. EHI is provided to employed workers (company employee) and their dependents and by many insurance companies (> 1,500 insurance companies in Japan). NHI is provided by municipalities for persons who are not provided with EHI (i.e. the self-employed, those working in agriculture, forestry or small businesses) and are under 74 years of age. Persons not covered by the EHI or NHI (i.e. for self-employed persons aged 75 years or older) are covered through LEHI provided on the prefecture-level.

From June to October every year, the NHI provides an annual health check-up at designated community centers and medical institutions for NHI-insured people aged 40 to 74. Participation is voluntary, and municipalities keep data on the results of the health check-ups. Each household is notified about the health check-up every year based on the city’s family register. Health check-ups include physical measurements and blood sample tests. For this study, Minamisoma City provided us with the anonymized health check-up result data for all participants from 2010 to 2017.

This study focuses on three non-communicable diseases as the main study outcomes which can be evaluated from blood parameter data covered by health check-up. Diabetes: defined as HbA1c of more than 6.5%. Hyperlipidemia: defined as low-density lipoprotein cholesterol (LDL) of more than 140 mg/dL; or high-density lipoprotein cholesterol (HDL) of less than 40 mg/dL; or triglyceride of more than 150 mg/dL. Hypertension: defined as systolic blood pressure (SBP) of more than 140 mm Hg; or diastolic blood pressure (DBP) of more than 90 mm Hg. These definitions were based on the major clinical guidelines and are widely used [29–31]. Diagnosis of the diseases based on these definitions was made on year-by-year basis.

### Data – evacuation scenario

The study participants were divided into five groups according to evacuation scenarios. Both voluntary and mandatory evacuees were included as evacuees. In the present study, evacuation was defined as the movement of people in 2011 from their place of residence at the ward level (administrative level below the municipality) in 2010. The focus on ward level was because the initial evacuation orders in the city were largely based on the ward level, and cultural regional differences are also observed at the ward level. The five evacuation groups defined in this study are as follows: The group that did not evacuate after the incident (‘no-evacuation’); the group that returned one year after the incident (‘return in 2012ʹ); the group that returned two to four years after the incident (‘return in 2013–15ʹ); the group that returned five to six years after the incident (‘return in 2016–17ʹ); and the group that remained evacuated at the time of the 2017 check-up (‘no-return’). Evacuation information was obtained from the evacuation database of Minamisoma City. As described above, the address on the residence certificate and the actual place of residence may differ after the accident. The definition of evacuation in this study and the evacuation database was based on this actual residential address. This is a unique database created by Minamisoma City to provide important administrative communications such as tax notices to evacuees, and is linked to administrative services such as health check-ups on an individual basis by the city. Details are provided elsewhere [32,33]. Although it was possible to determine whether a person was voluntarily or forcibly evacuated based on whether their place of residence was designated as a mandatory evacuation area or not, this difference could not be taken into account in analytical statistics because the number of participants with the target outcomes was too limited to conduct an analysis stratified by evacuation type. In this study, in addition to the anonymized health check-up result data, Minamisoma City provided us with the linked evacuation data.

### Analyses

First, to examine the possible selection bias, we compared the crude prevalence of the diseases in 2010 between those who also participated in the health check-ups in 2017 (i.e. those considered in the study) and those who did not participate (i.e. those excluded in this study). In addition, to assess whether there could be a difference in the impact on disease risks between voluntary evacuation and mandatory evacuation, we calculated the crude prevalence of the diseases of each group in 2010 and 2017 using the chi-square test.

Second, we estimated the annual age-adjusted prevalence of diabetes, hyperlipidemia, and hypertension from 2010 to 2017 in order to consider the different age distributions among the participants each year. As a first step, we estimated a simple logistic regression model for each disease in which the binomial data for each disease based on the health check-up data were used as a dependent variable, and year of the health check-up (as a categorical variable) and age at that time (as a continuous variable) were used as independent variables. The age-adjusted prevalence of each disease was then estimated by imputing the mean age of the participants in each corresponding year to the age variable in the estimated regression model. This is a common method to estimate predicted probabilities following confounder-adjusted logistic regression, known as the prediction at the means method [34].

To assess whether there was a linear trend in the prevalence of diabetes, hyperlipidemia, and hypertension from 2010 to 2017 after adjusting for age, we treated year of the health check-up as a continuous variable in the models, rather than a categorical variable. The significance of the coefficient of its linear trend (called p-for-trend) was assessed with the Wald test. This is a common method to evaluate dose-response effects in associations [35]. The regression models for these analyses were constructed separately for each disease and each evacuation scenario.

Third, we estimated the differences in the risk of diabetes, hyperlipidemia, and hypertension by evacuation scenario at the time of the 2017 health check-up (in reference to the no-evacuation group). Because of the possible selection bias at the individual level, we used inverse-probability weighting (IPW) to adjust for possible imbalances between the evacuation scenarios. IPW is a variant of the propensity scores matching, which can retain all observations [36], and allows for better confounding adjustment in observational studies [37,38]. In this context, the imbalances refer to the different distribution of baseline covariates for each evacuation scenario. To perform IPW, a multinomial logistic regression model was employed, where dependent variables were a binary variable of whether or not the participant had diabetes, hyperlipidemia, and hypertension (defined above) at the 2017 health check-up. Covariate used in the matching procedure were baseline data from 2010 health check results, such as physical measurements and blood sample tests (see resulting tables).

Balances in the distribution of baseline covariates between evacuation scenarios were assessed by estimating absolute standardized differences between the scenarios for each covariate and before and after matching [39]. Standardized differences quantify the difference in the means or proportions in units of covariates between the scenarios, and are expressed as percentages of the pooled standard deviations [40]. Imbalance reduction after matching was then assessed by comparing the absolute standardized differences of covariates before and after matching. An absolute standardized difference of 0% on a covariate indicates no residual bias for that covariate, and many literature uses a value of 10% or less as an indication of inconsequential imbalances [41–45]. The analysis was performed separately for each disease, and included people who did not have the disease at the 2010 health check-up. Odds ratios (OR) with 95% confidence intervals (CI) for each outcome of interest were estimated separately.

We did not consider information on individual radiation dose or residential air dose level as a possible confounder in the regression analyses. Although it is not a definitive conclusion, a previous study suggested that the effects of moderate- to low-dose irradiation on hypothyroidism and autoimmune thyroiditis may be transient and reversible [46]. Furthermore, prior research has shown that the radiation dose level in Fukushima Prefecture has not been high enough after the accident to have a physical impact on the general population [1–8], and this has been re-confirmed by the latest 2020 report of the United Nations Scientific Committee on the Effects of Atomic Radiation (UNSCEAR) [47], a special purpose body of the United Nations to assess the health effects of nuclear accidents. There is also literature suggesting that there was no statistically significant relationship between radiation dose and the risk of chronic diseases, including hypertension, in Fukushima Prefecture [14].

### Sensitivity analysis

We performed multiple sensitivity analyses to assess the robustness of our findings. First, we considered an augmented inverse-probability weighting (AIPW) method originally proposed in the missing data literature, which extends the IPW method by adding a regression term as an augmentation [48,49,50]. Second, the IPW analysis with the 2017 data only compares the disease risks between the evacuation scenarios as of 2017, and does not assess any earlier point in time. Therefore, sensitivity analysis was performed with the 2016 data with the purpose of confirming that the similar results can be obtained with data from an earlier time point than 2017. Data analysis were conducted using STATA/MP 16 .

## Results

Out of 5,528 participants in the 2010 health check-up, we included 1,837 participants who also participated in the 2017 health check-up. This included 1,166 females, accounting for 63.47% of the participants. There were no significant differences in the crude prevalence of the diseases between those included in the analysis and those excluded in 2017, with the exception of hyperlipidemia: for diabetes, there were positive cases in 157 participants (8.55%) who were included in the study, and 288 positive cases (10.09%) in those excluded (p = 0.08, chi-squared test); for hyperlipidemia, there were 680 positive cases (39.17%) in the those included and 1,104 (44.37%) in those excluded (p < 0.01); and for hypertension there were 347 positive cases (19.98%) in those included and 544 (21.85%) in those excluded (p = 0.14). Mean age at the 2010 health check-up was 60.75 with standard deviation (SD) of 5.55. The crude prevalence of the diseases was not significantly different between voluntary and mandatory evacuees, except for hyperlipidemia in 2010 and diabetes in 2017 (Table S1). Baseline demographic characteristics of the participants at the 2010 health check-up are presented in Table 1 by evacuation scenario, including the crude prevalence of the diseases for 2010 and 2017. There were no significant differences in the variables by evacuation scenarios, except for gender, age, smoking habits, and hypertension prevalence in 2017. A total of 849 (47.99%) participants had no experience of evacuation and 230 (13.00%) participants remained evacuated at the time of the 2017 check-up. (Table 1)

**Table 1.** Demographic characteristics of the study participants

Evacuation scenario	No-evacuation	Return in 2012	Return in 2013–15	Return in 2016–17	No-return
Number (n, %)**					
Female	523 (61.60)	300 (70.26)	89 (71.77)	82 (58.99)	136 (59.13)
Male	326 (38.40)	127 (29.74)	35 (28.23)	57 (41.01)	94 (40.87)
Physical measurements and blood sample test results in 2010					
Age (mean, SD)***	60.77 (5.53)	61.51 (5.09)	60.76 (5.68)	61.39 (5.45)	59.21 (5.83)
BMI (mean, SD)	23.20 (3.22)	23.10 (3.21)	23.11 (3.19)	22.92 (2.88)	23.23 (3.07)
SBP mmHg (mean, SD)	131.31 (15.97)	130.99 (17.26)	130.10 (16.69)	130.18 (15.35)	129.75 (14.80)
DBP mmHg (mean, SD)	78.93 (10.08)	78.11 (10.38)	78.14 (10.30)	79.01 (10.36)	78.14 (10.30)
HbA1c % (mean, SD)	5.44 (0.58)	5.45 (0.55)	5.44 (0.48)	5.53 (0.81)	5.47 (0.56)
Triglyceride mg/dL (mean, SD)	110.78 (69.04)	110.93 (95.92)	97.88 (45.82)	109.02 (90.31)	108.51 (92.32)
HDL mg/dL (mean, SD)	60.01 (14.46)	60.98 (14.68)	62.35 (13.94)	59.43 (12.78)	60.85 (15.02)
LDL mg/dL (mean, SD)	124.61 (30.00)	127.26 (29.86)	126.71 (31.06)	120.50 (30.83)	122.67 (28.82)
Daily smoker (n, %)*	107 (12.60)	40 (9.37)	17 (13.71)	13 (9.35)	40 (17.39)
Study outcomes in 2010 (n, %)					
Diabetes	24 (2.83)	20 (4.68)	2 (1.61)	7 (5.04)	8 (3.48)
Hyperlipidemia	321 (42.91)	170 (44.85)	39 (37.86)	50 (41.32)	73 (36.68)
Hypertension	230 (30.75)	133 (35.09)	33 (32.04)	37 (30.58)	53 (26.63)
Study outcomes in 2017 (n, %)					
Diabetes	69 (8.13)	32 (7.49)	7 (5.65)	16 (11.51)	27 (11.74)
Hyperlipidemia	302 (37.94)	163 (39.95)	45 (38.46)	53 (39.26)	91 (41.74)
Hypertension*	180 (22.61)	88 (21.52)	19 (16.24)	17 (12.59)	38 (17.43)

SD: standard deviation. BMI: body mass index. SBP: systolic blood pressure. DBP: diastolic blood pressure. HDL: high-density lipoprotein cholesterol. LDL: low-density lipoprotein cholesterol. * p < 0.05, ** p < 0.01, *** p < 0.001 (comparisons between evacuation scenarios with the ANOVA test for continuous and the chi-squared test for categorical variable).

**Figure 1.**
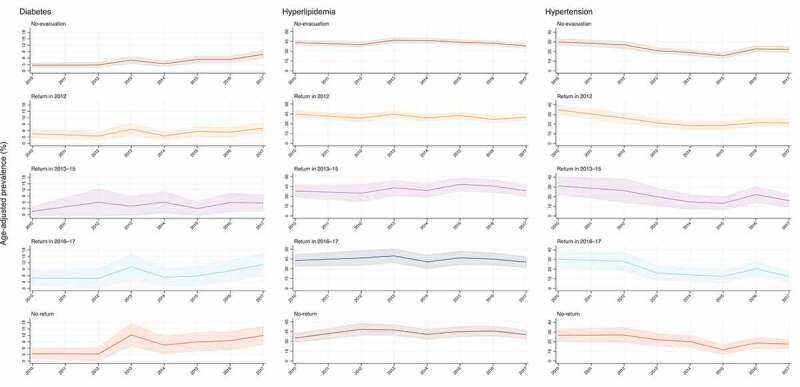
Annual age-adjusted prevalence of diabetes, hyperlipidemia, and hypertension by evacuation scenarios

The annual age-adjusted prevalence of each disease is plotted in Figure 1 by evacuation scenario. Regardless of evacuation scenario, diabetes prevalence showed an increasing trend over time (although statistical significance was only observed for the no-evacuation and no-return groups with p-for-trend < 0.05); hyperlipidemia prevalence showed no remarkable change; and hypertension prevalence showed a decreasing trend (all scenarios were significant with p-for-trend < 0.05) (Table 2).

**Table 2.** Odds ratios for the diseases risks per one year increase from 2010 to 2017 across evacuation scenarios, adjusting for age

	Odds ratio	95% CI	P-value
Diabetes			
No-evacuation	1.17	1.10–1.24	<0.001
Return in 2012	1.07	0.99–1.15	0.09
Return in 2013–15	1.13	0.96–1.34	0.15
Return in 2016–17	1.12	0.99–1.26	0.06
No-return	1.18	1.07–1.29	<0.01
Hyperlipidemia			
No-evacuation	0.98	0.96–1.01	0.13
Return in 2012	0.97	0.94–1.00	0.09
Return in 2013–15	1.03	0.96–1.10	0.41
Return in 2016–17	0.99	0.93–1.05	0.67
No-return	1.02	0.98–1.08	0.33
Hypertension			
No-evacuation	0.93	0.90–0.96	<0.001
Return in 2012	0.90	0.87–0.94	<0.001
Return in 2013–15	0.89	0.82–0.96	<0.01
Return in 2016–17	0.86	0.80–0.93	<0.001
No-return	0.91	0.85–0.96	<0.01

CI: confidence intervals. Diabetes: HbA1c of more than 6.5%. Hyperlipidemia: low-density lipoprotein cholesterol of more than 140 mg/dL; or high-density lipoprotein cholesterol of less than 40 mg/dL; or triglyceride of more than 150 mg/dL. Hypertension: systolic blood pressure of more than 140 mm Hg; or diastolic blood pressure of more than 90 mm Hg.

Diabetes: HbA1c of more than 6.5%. Hyperlipidemia: low-density lipoprotein cholesterol of more than 140 mg/dL; or high-density lipoprotein cholesterol of less than 40 mg/dL; or triglyceride of more than 150 mg/dL. Hypertension: systolic blood pressure of more than 140 mm Hg; or diastolic blood pressure of more than 90 mm Hg. Logistic regression models were used to predict the prevalence, while holding age at the mean values among the whole participants (60.75): Solid lines are point estimates and shaded areas show 95% confidence intervals.

**Figure 2.**
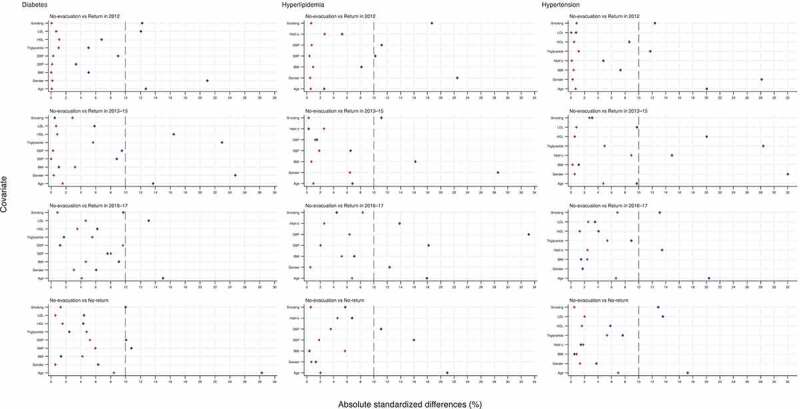
Absolute standardized differences for covariates in means before and after weighting for each comparison of evacuation scenarios in reference to no-evacuation

All absolute standardized differences after the IPW using the propensity scores were < 10%, indicating inconsequential imbalances of the baseline covariates for each comparison of evacuation scenarios with no-evacuation (Figure 2). The IPW analyses demonstrated that for each disease, evacuation scenarios were not significantly associated with the disease risk at the 2017 health check-up (Table 3). Although statistical significance was observed in the comparison between the no-evacuation and no-return groups for hypertension (0.94, 95% CI 0.89–1.00, p < 0.05), this disappeared in the sensitivity analysis (Table S2).

**Table 3.** Comparative risk of diabetes, hyperlipidemia, and hypertension at the 2017 health check-ups across evacuation scenarios in reference to the no-evacuation group

	Odds ratio	95% CI	P-value
Diabetes			
Return in 2012	0.98	0.96–1.01	0.29
Return in 2013–15	1.00	0.95–1.04	0.90
Return in 2016–17	1.01	0.96–1.07	0.71
No-return	1.04	0.99–1.09	0.16
Hyperlipidemia			
Return in 2012	1.02	0.95–1.10	0.58
Return in 2013–15	0.97	0.87–1.09	0.64
Return in 2016–17	1.07	0.94–1.23	0.28
No-return	0.98	0.89–1.06	0.59
Hypertension			
Return in 2012	1.01	0.96–1.07	0.73
Return in 2013–15	0.99	0.90–1.08	0.78
Return in 2016–17	0.94	0.87–1.01	0.07
No-return	0.94	0.89–1.00	<0.05

CI: confidence intervals. Diabetes: HbA1c of more than 6.5%. Hyperlipidemia: low-density lipoprotein cholesterol of more than 140 mg/dL; or high-density lipoprotein cholesterol of less than 40 mg/dL; or triglyceride of more than 150 mg/dL. Hypertension: systolic blood pressure of more than 140 mm Hg; or diastolic blood pressure of more than 90 mm Hg.

Diamond (blue) and cross (red) symbols refer to the before- and after-weighting, respectively. Diabetes: HbA1c of more than 6.5%. Hyperlipidemia: low-density lipoprotein cholesterol of more than 140 mg/dL; or high-density lipoprotein cholesterol of less than 40 mg/dL; or triglyceride of more than 150 mg/dL. Hypertension: systolic blood pressure of more than 140 mm Hg; or diastolic blood pressure of more than 90 mm Hg.

## Discussion

Looking at temporal changes in non-communicable diseases, this study found that the risk of diabetes increased over time, and that of hypertension tended to decrease. There was no statistical evidence suggesting a change in the hyperlipidemia risk. These trends were similar regardless of the evacuation scenario.

A recent systematic review by Gohardehi et al. (2020) assessed the prevalence of diabetes and hypertension in populations affected by natural disasters globally and found evidence for increased risks of these diseases post-disaster [51], although the follow-up periods of the study populations were limited to short-term periods of one to several years. Existing literature suggests that, in the aftermath of a disaster, a variety of socioeconomic and psychological factors can lead to the recurrence of previously controlled diabetes and hypertension, or to the further progression of these diseases in people with pre-hypertension or pre-diabetes [51–56]. This may contribute to the increased prevalence of these diseases among affected populations. Other systematic reviews have noted similar results [57,58], suggesting that populations affected by natural disasters are at higher risk for diabetes and hypertension than the general population.

Similar to these previous studies from different disaster settings, studies in the context of the Great East Japan Earthquake and the Fukushima nuclear accident have also reported increased diabetes and hypertension risks among affected populations over the first few years post-disaster [13–17]. Our findings are consistent with these previous studies for diabetes, but not for hypertension. The major difference observed in hypertension trends between these previous studies and the present study may be explained by the fact that the presence or absence of antihypertensive drug use could not be taken into account in our definition of hypertension due to insufficient data in the present study. The declining trend of hypertension risk observed in this study may reflect that although there was an increase in the number of people requiring antihypertensive treatment after the accident, as pointed out in previous studies [16,17,51–55,57,58,59,60], the blood pressure of those people was well controlled. In fact, adequate control of hypertension is possible because blood pressure can be monitored daily using a home sphygmomanometer after a disaster [61]. On the other hand, diabetes is difficult to control after a disaster; most available glucose monitoring requires invasive procedures, and self-monitoring is not widely accepted by patients [62–66].

The same logic may apply to hyperlipidemia. Although previous studies conducted outside Japan on post-disaster hyperlipidemia are limited, the study by Fonesca et al. (2009) and Gautam et al. (2013) noted elevated LDL cholesterol levels in the affected populations after the 2005 Hurricane Katrina [53,67], although they were short-term studies with about two years of follow-up. In the context of the Great East Japan Earthquake and the Fukushima nuclear accident, previous studies showed that the hyperlipidemia prevalence tended to increase some years after the earthquake and accident in Fukushima Prefecture [14,17,68,69], although our study did not show a significant increase. One of the possible reasons for this is that the present study did not consider if patients were receiving treatment. The absence of an increased risk of hyperlipidemia in this study may indicate that cholesterol control was somewhat successful.

In addition, the IPW analysis revealed that the disease risks in 2017 did not differ significantly between different evacuation scenarios, which is a thought-provoking result. Previous results on whether evacuation itself was an independent driving factor for an increase in diabetes and hypertension vary from study to study [13–17], suggesting that region-specific factors played an important role in the health effects of the evacuation. Our findings might reflect the high awareness of lifestyle-related disease control in the general public post-disaster, including diabetes, hypertension, and hyperlipidemia in Minamisoma City – the possible positive impact of public awareness of lifestyle risk factors for chronic diseases has been widely discussed [70–72]. Rather than overemphasizing the health risks of evacuation, we would like to emphasize that there is much room to reduce the health impacts of evacuation by strengthening the disaster resilience of local health systems. This can be successful, for example, through disease-specific and targeted measures in communities and the promotion of healthy lifestyles (e.g. weight management, sleep behaviors, balanced diets) at the individual level [58,73,74].

Yet another explanation is that complex confounding factors may be involved between evacuation and long-term health risks, and the implications of the health effects of evacuation may vary depending on factors that can be adjusted within each study, potentially leading to different observations to date. For example, the health effects of evacuation may largely depend on upstream social determinants of health, such as socioeconomic and political contexts [75], including local welfare systems and disaster risk management. These are likely unobservable in conventional epidemiological studies which tend to rely on biomedical data.

Not many studies have assessed the long-term health effects of post-disaster evacuation in a localized manner. Further research would be helpful on the regional level to understand the long-term health status of evacuees, assess the relationship between evacuation and health status, and evaluate what community-specific factors may have exacerbated or minimized any effects of evacuation on health in disaster-affected areas. These types of studies could help improve evidence to leverage global, national, and community efforts to reduce the health risks of evacuation as much as possible in any future disasters requiring evacuation.

## Limitations

There are several limitations to this study. First, participation in the public health check-up is voluntary and is only available to residents aged 40–74 who are covered by NHI. This population is self-employed or in other occupations where health insurance is not provided through employers (e.g. agriculture, forestry, fishing). Therefore, the results of this study may be biased, and generalization to the broader population is likely to be limited, especially to different occupations and age groups. In addition, self-selection bias (i.e. people decide for themselves whether or not to participate in health check-ups) may have affected the results. For example, if people who are more concerned about their health risks are more likely to participate in health check-ups, and more likely to exercise and eat a healthy diet, the risk of lifestyle-related diseases in the population analyzed in this study may be underestimated compared to the entire population of the same age group in Minamisoma City [76,77]. Similarly, if there are any differences in trends of health concerns (and subsequent health check participation) between non-evacuees and evacuees, the risk comparison by evacuation scenarios in this study may be biased. In addition, the study included people who underwent both the 2017 and 2010 health check-ups, which accounted for 21% of the 2010 health check-ups participants. In other words, 79% did not have a health check-up in 2017. The cause for this loss to follow-up is unknown. It is possible that participants may have been older than their eligible age for screening (up to 74 years old) during the period, or that they may have been evacuated and had their residence certificate transferred to another municipality, or may have just moved in, or may have just happened to miss the health check-up in 2017, or that they were not healthy enough to visit a health center or medical institution to participate in the health check-up. For any of these reasons, it is possible that those regularly participating in the health checks (and included in this study) may have differed from those lost to follow-up, and these results should be interpreted cautiously. Further, although it is well known that there are age and gender differences in disease risks post-accident [78], age- or gender-specific assessment was not possible in this study because of the limited number of participants, while they were used as adjustment variables in the present study. Other limitations of this study include that it was not possible to analyze by type of evacuation (voluntary evacuation or mandatory evacuation) or by type of accommodation post-evacuation (temporary housing and other types of accommodation) [79]. Although there were some slightly statistically significant differences in the crude prevalence of the diseases, no major differences were observed between voluntary evacuation and mandatory evacuation at both the 2010 and 2017 health check-ups.

## Conclusion

Although caution is necessary as this study was limited to residents covered by NHI (who form a particular occupational makeup), this research provides new findings on long-term trends in non-communicable diseases. Regardless of evacuation scenario, there was a statistical evidence suggesting an upward and a downward trend in diabetes and hypertension from 2010 to 2017, respectively, while hyperlipidemia showed no remarkable change. In addition, after adjustment for baseline covariates with IPW, the disease risks as of 2017 did not differ significantly among people with different evacuation scenarios in Minamisoma City. Our findings highlight the need to assess the health effects of evacuations in a more localized manner. Further research in this area can help to strengthen community preparedness and government management of future disasters that require mass evacuation.

## Supplementary Material

Supplemental Material

## Paper context

Japan’s Fukushima Daiichi nuclear power plant in 2011 resulted in a large number of evacuees. Using annual health check-ups from 2010 to 2017 administered by Minamisoma City, one of the closest municipalities in Fukushima Prefecture to the power plant, we demonstrated that the risks of diabetes, hypertension, and hyperlipidemia as of 2017 did not differ significantly among people with different evacuation scenarios.
