# Supplementary material for: Comparative risk assessment of non-communicable diseases by evacuation scenario– a retrospective study in the 7 years following the Fukushima Daiichi nuclear power plant accident
Source: Glob Health Action. 2021 Jun 1;14(1):1918886. doi: 10.1080/16549716.2021.1918886 (PMC8172221; doi:10.1080/16549716.2021.1918886)
Supplement: Supplemental Material [file ZGHA_A_1918886_SM0714.docx]

**Supplementary table s1: Crude prevalence of the diseases by evacuation type.**

| Study outcomes in 2010 (n, %) | Voluntary evacuees | Mandatory evacuees |
| --- | --- | --- |
| Diabetes | 22 (3.9) | 15 (4.21) |
| Hyperlipidemia* | 220 (44.53) | 112 (36.36) |
| Hypertension | 168 (34.01) | 88 (28.57) |
| Study outcomes in 2017 (n, %) |  |  |
| Diabetes* | 42 (7.45) | 40 (11.24) |
| Hyperlipidemia | 214 (39.78) | 138 (40.59) |
| Hypertension | 107 (19.85) | 55 (16.18) |

* p<0.05 (comparisons between voluntary evacuees and mandatory evacuees with the Chi-squared test).

**Supplementary table s2: Sensitivity analyses – comparative risk of diabetes, hyperlipidemia, and hypertension at the 2017 health check-ups across evacuation scenarios in reference to the no-evacuation group: (A) using an augmented inverse probability weighting method, (B) follow-up to 2016.**

| (A) | Odds ratio | 95% CI | P-value | (B) | Odds ratio | 95% CI | P-value |
| --- | --- | --- | --- | --- | --- | --- | --- |
| Diabetes |  |  |  | Diabetes |  |  |  |
| Return in 2012 | 0.98 | 0.95–1.01 | 0.18 | Return in 2012 | 1.01 | 0.98–1.03 | 0.55 |
| Return in 2013–15 | 0.99 | 0.95–1.04 | 0.71 | Return in 2013–16 | 1.02 | 0.97–1.07 | 0.42 |
| Return in 2016–17 | 1.01 | 0.96–1.05 | 0.84 | No-return | 1.03 | 1.00–1.07 | 0.06 |
| No-return | 1.03 | 0.99–1.08 | 0.15 |  |  |  |  |
| Hyperlipidemia |  |  |  | Hyperlipidemia |  |  |  |
| Return in 2012 | 1.01 | 0.94–1.09 | 0.80 | Return in 2012 | 1.00 | 0.93–1.07 | 0.93 |
| Return in 2013–15 | 0.98 | 0.88–1.09 | 0.65 | Return in 2013–16 | 1.12 | 1.00–1.26 | 0.05 |
| Return in 2016–17 | 1.07 | 0.94–1.21 | 0.31 | No-return | 1.06 | 0.98–1.15 | 0.14 |
| No-return | 0.98 | 0.90–1.07 | 0.64 |  |  |  |  |
| Hypertension |  |  |  | Hypertension |  |  |  |
| Return in 2012 | 0.99 | 0.94–1.05 | 0.80 | Return in 2012 | 0.97 | 0.92–1.02 | 0.23 |
| Return in 2013–15 | 0.99 | 0.90–1.10 | 0.91 | Return in 2013–16 | 0.98 | 0.90–1.06 | 0.61 |
| Return in 2016–17 | 0.94 | 0.88–1.00 | 0.05 | No-return | 0.98 | 0.93–1.04 | 0.46 |
| No-return | 0.95 | 0.90–1.00 | 0.07 |  |  |  |  |

CI: confidence intervals. Diabetes: HbA1c of more than 6.5%. Hyperlipidemia: low-density lipoprotein cholesterol of more than 140 mg/dL; or high-density lipoprotein cholesterol of less than 40 mg/dL; or triglyceride of more than 150 mg/dL. Hypertension: systolic blood pressure of more than 140 mm Hg; or diastolic blood pressure of more than 90 mm Hg. The evacuation scenarios for (B) were modified in consideration of the number of participants with diseases. Absolute standardized differences for covariates in means before and after weighting for (A) and (B) are presented in Supplementary figures s1 and s2, respectively.

**Supplementary figure s1: Sensitivity analyses ­using an augmented inverse probability weighting method – absolute standardized differences for covariates in means before and after weighting for each comparison of evacuation scenarios.**

Diamond (blue) and cross (red) symbols refer to the before- and after-weighting, respectively. Diabetes: HbA1c of more than 6.5%. Hyperlipidemia: low-density lipoprotein cholesterol of more than 140 mg/dL; or high-density lipoprotein cholesterol of less than 40 mg/dL; or triglyceride of more than 150 mg/dL. Hypertension: systolic blood pressure of more than 140 mm Hg; or diastolic blood pressure of more than 90 mm Hg.

**Supplementary figure s2: Sensitivity analyses ­with the follow-up to 2016 – absolute standardized differences for covariates in means before and after weighting for each comparison of evacuation scenarios.**

Diamond (blue) and cross (red) symbols refer to the before- and after-weighting, respectively. The evacuation scenarios were modified in consideration of the number of participants with diseases. Diabetes: HbA1c of more than 6.5%. Hyperlipidemia: low-density lipoprotein cholesterol of more than 140 mg/dL; or high-density lipoprotein cholesterol of less than 40 mg/dL; or triglyceride of more than 150 mg/dL. Hypertension: systolic blood pressure of more than 140 mm Hg; or diastolic blood pressure of more than 90 mm Hg.
